# Transcriptional Profiling of Biofilm Regulators Identified by an Overexpression Screen in *Saccharomyces cerevisiae*

**DOI:** 10.1534/g3.117.042440

**Published:** 2017-07-03

**Authors:** Gareth A. Cromie, Zhihao Tan, Michelle Hays, Amy Sirr, Eric W. Jeffery, Aimée M. Dudley

**Affiliations:** * Pacific Northwest Research Institute, Seattle, Washington 98122; † Institute of Medical Biology, Agency for Science, Technology and Research, Singapore 138648; ‡ Molecular and Cellular Biology Program, University of Washington, Seattle, Washington 98195

**Keywords:** biofilm, transcriptional regulation, overexpression, colony morphology

## Abstract

Biofilm formation by microorganisms is a major cause of recurring infections and removal of biofilms has proven to be extremely difficult given their inherent drug resistance . Understanding the biological processes that underlie biofilm formation is thus extremely important and could lead to the development of more effective drug therapies, resulting in better infection outcomes. Using the yeast *Saccharomyces cerevisiae* as a biofilm model, overexpression screens identified *DIG1*, *SFL1*, *HEK2*, *TOS8*, *SAN1*, and *ROF1/YHR177W* as regulators of biofilm formation. Subsequent RNA-seq analysis of biofilm and nonbiofilm-forming strains revealed that all of the overexpression strains, other than *DIG1* and *TOS8*, were adopting a single differential expression profile, although induced to varying degrees. *TOS8* adopted a separate profile, while the expression profile of *DIG1* reflected the common pattern seen in most of the strains, plus substantial *DIG1*-specific expression changes. We interpret the existence of the common transcriptional pattern seen across multiple, unrelated overexpression strains as reflecting a transcriptional state, that the yeast cell can access through regulatory signaling mechanisms, allowing an adaptive morphological change between biofilm-forming and nonbiofilm states.

Many opportunistic human pathogens form highly structured, multicellular communities called biofilms, which are a key factor in persistent infections (Costerton *et al.* 1999; Donlan and Costerton 2002; Fanning and Mitchell 2012). Biofilms are a major cause of medical-device associated infections (Costerton *et al.* 1999; Donlan and Costerton 2002; Darouiche 2004), chronic nonhealing of wounds (Mancl *et al.* 2013; Scali and Kunimoto 2013), and infections of the oral (Mancl *et al.* 2013), respiratory (Kobayashi 2005) and urinary tract surfaces (Tenke *et al.* 2012). The ability of biofilms to adhere to organic and inorganic surfaces as well as their increased drug resistance makes them a pressing clinical problem (Costerton *et al.* 1999; Donlan and Costerton 2002; Ramage *et al.* 2010). The transition from a planktonic, unicellular lifestyle to a sessile, multicellular lifestyle requires the coordinated activation and repression of numerous biological processes. While some of these pathways have been elucidated, many remain poorly characterized (Finkel and Mitchell 2011; Fanning and Mitchell 2012). A better understanding of the molecular mechanisms required for biofilm formation is needed to guide the development of drug therapies that specifically target biofilms, an area that is largely underdeveloped (Ramage *et al.* 2010; Pierce and Lopez-Ribot 2013).

The yeast *Saccharomyces cerevisiae* is an established model for the study of biofilm formation. While most *S. cerevisiae* strains form smooth, unstructured colonies on solid media, some strains are able to form highly structured “fluffy” colonies. Fluffy *S. cerevisiae* colonies not only visually resemble bacterial and fungal biofilms, but also share their structural and functional characteristics, including the presence of an extracellular matrix (Kuthan *et al.* 2003; Karunanithi *et al.* 2010; Vachova *et al.* 2011), localized expression of drug efflux pumps (Vachova *et al.* 2011), the use of intercellular communication (Vopalenska *et al.* 2010), reduced penetration of chemicals into the interior of the colony (Vachova *et al.* 2011), and increased adherence to inorganic surfaces (Reynolds and Fink 2001).

A number of detailed genetic screens have been performed to elucidate the genes involved in colony morphology (Granek and Magwene 2010; Ryan *et al.* 2012; Voordeckers *et al.* 2012; Taylor and Ehrenreich 2015). Since the commonly used FY lab strain background (Winston *et al.* 1995) is unable to adopt the fluffy colony morphology, these studies have been performed with other strains that are able to form structured colonies. Results from these studies demonstrated that biofilm formation is finely tuned to the environment, with the “filamentation” mitogen-activated protein kinase (MAPK) (Granek and Magwene 2010; Voordeckers *et al.* 2012), Ras-cAMP-PKA (Granek and Magwene 2010), target-of-rapamycin (TOR) (Voordeckers *et al.* 2012), and high osmolarity glycerol (Voordeckers *et al.* 2012) signaling pathways all playing important roles in modulating colony morphology. Notably, the filamentation MAPK pathway regulates genes, such as the adhesin *FLO11*, that appear to be required for complex colony formation, while pseudohyphal growth itself is not essential for this phenotype (Sťovíček *et al.* 2010).

Another approach to understanding biofilm formation has been the comparison of wild yeast strains in their biofilm and nonbiofilm forms. Because naturally occurring fluffy wild strains can switch to a nonbiofilm “smooth” colony-forming state at relatively high rates during the process of domestication in the laboratory (Kuthan *et al.* 2003), it is possible to isolate smooth and fluffy forms of the same strain. These studies have uncovered metabolic differences between fluffy strains and their smooth counterparts, including the differential expression of genes involved in metabolism and the transport of carbohydrates, vitamins, and amino acids (Kuthan *et al.* 2003; Sťovíček *et al.* 2014). A fluffy strain (F45) studied in our laboratory also switched between fluffy and smooth colony morphologies at rates much higher than that of spontaneous mutations (Tan *et al.* 2013). In this strain, the mechanism underlying the switch was the gain and loss of individual chromosomes. Because increased copy number of only a subset of the 16 *S. cerevisiae* chromosomes could induce the phenotypic change, we hypothesized that the switch was caused by dosage changes of specific genes on those chromosomes. Consistent with this model, a genetic screen for individual genes on one of these chromosomes (XVI) whose modest overexpression was sufficient to cause the fluffy-to-smooth transition, identified *DIG1* (Tan *et al.* 2013), which encodes a transcriptional repressor in the filamentation MAPK pathway (Cook *et al.* 1996).

Here, we extend our screen genome-wide and identify five additional genes whose overexpression causes fluffy F45 colonies to become smooth. We then use RNA-seq to characterize the transcriptional profiles of the smooth overexpression strains relative to the original F45 strain and look for functional enrichment among the most strongly induced and repressed genes. Several of our results support a model in which regulated changes between a small number of transcriptional states underlie changes in colony morphology. First, all six of the genes identified in our overexpression screens encode regulatory proteins. Second, five of these genes, when overexpressed, induce a single transcriptional response, along with a change in colony morphology, suggesting they influence a common signaling network. Third, the transcriptional responses that we observe include genes, such as *FLO11*, that are known to effect changes in colony morphology.

## Materials and Methods

### Yeast strains and media

Unless noted, standard media and methods were used for growth and genetic manipulation of yeast (Rose 1990). The strains of *S. cerevisiae* used in this study are listed in Table 1.

**Table 1:** Strains used in this study

Strain Name	Progenitor	Genotype	Source
YO1853	F45	*MAT*a *ho∆0*::*hphMX6*, *SPS2*:*EGFP*:*natMX4*, unmapped serine auxotroph, [pRS41K]	Strain (Tan *et al.* 2013), plasmid (Taxis and Knop 2006)
YO1773	F45	*MAT*a *ho∆0*::*hphMX6*, *SPS2*:*EGFP*:*natMX4*, unmapped serine auxotroph, [DIG1-pFA6a-KanMX4]	Tan *et al.* (2013)
YO1829	F45	*MAT*a *ho∆0*::*hphMX6*, *SPS2*:*EGFP*:*natMX4*, unmapped serine auxotroph, [SAN1-p5472]	Ho *et al.* (2009)
YO1832	F45	*MAT*a *ho∆0*::*hphMX6*, *SPS2*:*EGFP*:*natMX4*, unmapped serine auxotroph, [TOS8-p5472]	Ho *et al.* (2009)
YO1835	F45	*MAT*a *ho∆0*::*hphMX6*, *SPS2*:*EGFP*:*natMX4*, unmapped serine auxotroph, [ROF1-p5472]	Ho *et al.* (2009)
YO1845	F45	*MAT*a *ho∆0*::*hphMX6*, *SPS2*:*EGFP*:*natMX4*, unmapped serine auxotroph, [SFL1-p5472]	Ho *et al.* (2009)
YO1849	F45	*MAT*a *ho∆0*::*hphMX6*, *SPS2*:*EGFP*:*natMX4*, unmapped serine auxotroph, [HEK2-p5472]	Ho *et al.* (2009)
YO2111	F45	*MAT*a *ho∆0*::*hphMX6*, *SPS2*:*EGFP*:*natMX4*, unmapped serine auxotroph, [SAN1(R280A)-pRS41k]	Ho *et al.* (2009)
YO780	F13	*MAT*α *ho∆0*::*hphMX6*, *SPS2*:*EGFP*:*natMX4*	This study
YO1737	F13	*MAT*α *ho∆0*::*hphMX6*, *SPS2*:*EGFP*:*natMX4*, *dig1Δ*::*kanMX4*	This study
YO1898	F13	*MAT*α *ho∆0*::*hphMX6*, *SPS2*:*EGFP*:*natMX4*, *san1Δ*::*kanMX4*	This study
YO1902	F13	*MAT*α *ho∆0*::*hphMX6*, *SPS2*:*EGFP*:*natMX4*, *tos8Δ*::*kanMX4*	This study
YO1908	F13	*MAT*α *ho∆0*::*hphMX6*, *SPS2*:*EGFP*:*natMX4*, *rof1Δ*::*kanMX4*	This study
YO1914	F13	*MAT*α *ho∆0*::*hphMX6*, *SPS2*:*EGFP*:*natMX4*, *sfl1Δ*::*kanMX4*	This study
YO1910	F13	*MAT*α *ho∆0*::*hphMX6*, *SPS2*:*EGFP*:*natMX4*, *hek2Δ*::*kanMX4*	This study

### Overexpression screen

In order to perform the overexpression screen using the MoBY plasmid library (Ho *et al.* 2009), we first created pools of plasmids by pinning the master library from 96-well plates to selective (G418) LB Lennox Omnitrays and scraping the colonies from 10 such pinned plates into each pool pellet. Each pool pellet of ∼3 g of cells was split over four Qiafilter Plasmid Maxi Prep columns (Qiagen) per the manufacturer’s protocol.

With an assumed 1000 plasmids per pool, screening 4600 transformants was expected to give us a 99% chance of recovering each plasmid at least once. Based on this, we screened five bioassay trays (YPD with 2% glucose and G418 for plasmid selection) per pool, plating 1000–1500 CFU per tray. Transformations were performed using standard LiAc/DMSO methods. Colony morphology was examined after 4 d of colony growth. Transformants with completely smooth or intermediate, *i.e.*, more smooth than the F45 parent, phenotypes were picked. The frequency of smooth colonies following pool transformation was low (<5%) in all the pools. Transformation using a negative vector control, AB352 (pFA6a, Addgene), did not produce any smooth colonies.

Plasmid DNA was prepared from the smooth strains by standard methods (Hoffman and Winston 1987) and the identity of the overexpressed gene in each strain was then identified by PCR amplification and conventional ABI sequencing of the UPTAG and DNTAG barcodes (Ho *et al.* 2009). Strains with failed sequencing or inconsistent barcodes were removed from further consideration, leaving a set of 292 strains harboring 259 distinct plasmids.

Among the final set of plasmids, only 18 were identified more than once, leaving 241 singletons. The large proportion of single hits could have been due to a high false positive rate and/or failure to saturate the screen. To distinguish between these possibilities, we constructed a new smaller pool (a “singleton” pool) that contained 91 plasmids that came up in the list of single hits. The relative number of smooth *vs.* fluffy colonies resulting from transformation with this singleton pool would help distinguish if the false positive rate was high or if the screen was undersaturated. The transformation plates were almost entirely fluffy with <1% smooths in ∼3000 CFU. This suggested that the singletons observed in the screen were largely false positives, rather than reflecting screen undersaturation.

The 18 plasmids observed more than once in the initial screen were retested by individual transformations of each plasmid into F45, followed by screening for colony morphology. Plasmids that induced a phenotype change were further verified to ensure they contained the correct ORF, based on restriction digest pattern and size confirmation by PCR. Only five genes were verified as positive: *SAN1*, *TOS8*, *YHR177W*, *HEK2*, and *SFL1*. This included all three of the genes isolated >2 times, but only 2 of the 15 genes observed twice.

### RNA preparation and sequencing

After 4 d of growth on YPD (with 2% glucose plus G418 to maintain plasmid selection) plates at 30°, whole colonies, arrayed in a “checkerboard” pattern (Ruusuvuori *et al.* 2014), were harvested by scraping off the surface of the agar plate. To obtain sufficient amounts of RNA, three to five colonies were pooled for each sample, with four replicate samples of each strain.

Following extraction by hot acid phenol (Collart and Oliviero 2001), total RNA from the pooled colonies was quantified by Bioanalyzer (Agilent). Five micrograms of total RNA for each sample was then processed using the Tru-Seq stranded mRNA kit (Illumina) following manufacturer instructions. Individual sequencing libraries were pooled and analyzed by paired-end, 51 nucleotide read sequencing in one lane of an Illumina HiSeq 2000.

### Read-pair alignment

Read-pair alignment for RNA-seq data was carried out against the S288c reference (R64-1-1), with the FASTA and GFF files extended to include noncoding RNAs (ncRNAs) and genes present in F45, but absent in S288c, as described in Cromie *et al.* (2017), using Bowtie2 (version 2.1.0) (Langmead and Salzberg 2012) with the parameters [-N 1 -I 50 -X 450 -p 6–reorder -x -S] and allowing one mismatch per read. For each strain, read alignments were converted to gene counts using featureCounts (version 1.4.0) in the Subread package (Liao *et al.* 2014), with the parameters [-a -o -t gene –g ID –s 2 -T 1 -p -P -d 50 -D 450]. Reads were not filtered based on mapping quality, and thus we have been cautious in our interpretation of counts of genes that have paralogs with similar sequences, or which contain large regions of low sequence complexity. Read sequences and gene count tables are available from the Gene Expression Omnibus under accession GSE98079. Data for the control strain (empty vector) and the *DIG1* overexpression strain have been previously published (Cromie *et al.* 2017) (GSE85843).

### Differential expression analysis

Analysis of differential gene expression was carried out using edgeR [v. 3.6.8] (Robinson *et al.* 2010) based on the tables of raw counts produced by featureCounts (*Materials and Methods*). The table of counts was split into two subtables, with the first consisting of ORFs present in the S288c reference genome (genes with systematic names beginning with “Y”) and the second consisting of the novel F45 genes and ncRNAs. Library sizes were normalized using calcNormFactors, and dispersion parameters were estimated using the estimateGLMTrendedDisp and estimateGLMTagwiseDisp commands. To identify genes differentially expressed between overexpression strains and the F45 empty-vector control, we conducted pairwise testing using the glmFit and glmLRT commands, with a *P*-value cutoff of 0.01 (after Benjamini & Hochberg multiple hypothesis correction, *i.e.*, false discovery rate). Only nuclear-encoded ORFs, present in the reference genome, with median basal expression of at least 1 read per million reads in the F45 empty-vector control were included when plotting log_2_ fold changes on scatterplots, carrying out factor analysis, and calculating regression parameters or correlation coefficients.

### Functional enrichment of gene lists

Functional enrichment of *S. cerevisiae* gene lists was performed using g:Profiler (http://biit.cs.ut.ee/gprofiler/). Holm-Bonferroni corrected enrichment *P*-values <0.05 were accepted, with moderate hierarchical filtering.

### Factor analysis

Factor analysis was carried out using the fa command in the “psych” package of R (version 3.1.1 (R Development Core Team 2014)), with a single factor specified and default parameters. Choice of a single factor was determined from scree and parallel analysis using the nScree command (package “nFactors”). Loadings for the single common factor were calculated using only nuclear-encoded ORFs with median basal expression of at least 1 read per million reads in F45. The loadings were: *DIG1* = 0.731, *SAN1* = 0.883, *TOS8* = 0.206, *ROF1/YHR177W* = 0.812, *SFL1* = 0.894, and *HEK2* = 0.918. Significant nonzero values on the common factor were identified using the general linear model functionality of edgeR (Robinson *et al.* 2010) and applying these loadings (normalized to sum to 1) as the “contrast” terms in the glmLRT command with a *P*-value cutoff of 0.01 (after Benjamini and Hochberg multiple hypothesis correction). Significant nonzero values for the residual *DIG1* profile were identified as the residual from a linear regression between the *DIG1* profile and the common factor. The “contrast” terms for the common factor, multiplied by the regression coefficient, were subtracted from the contrast terms used to calculate the full *DIG1* profile to give the contrast terms producing the residual. The variance explained by the common factor for each overexpression profile was calculated as the variance of the overexpression profile times the communality value from the factor analysis. The communality values were: *DIG1* = 0.534, *SAN1* = 0.780, *TOS8* = 0.042, *ROF1/YHR177W* = 0.659, *SFL1* = 0.799, and *HEK2* = 0.842.

### Data availability

The datasets generated during and/or analyzed in the current study are available in the Gene Expression Omnibus (GEO) under GSE98079.

## Results

### A genetic screen for modulators of biofilm formation

The fact that increased copy number of several chromosomes could modulate fluffy colony morphology (Tan *et al.* 2013) suggested that increased copy number of other genes might regulate the trait in the same way as *DIG1*. To identify such modulators, we performed a large-scale overexpression screen for genes that reduced or eliminated the complex structure of the fluffy colonies. Briefly, we transformed the fluffy, euploid version of F45 from our previous study (Tan *et al.* 2013) with the MoBY plasmid collection, a low copy number (CEN) plasmid library containing 4981 individual ORFs under the transcriptional control of their native promoters (Ho *et al.* 2009). A set of 292 strains harboring 259 distinct plasmids were initially identified as having reduced colony morphology. Only 18 of these plasmids were identified more than once, suggesting either a high frequency of false positives or incomplete library coverage. Since retesting a subset of the singletons indicated that most were false positives (*Materials and Methods*) we proceeded with only the multiply-hit plasmids. The high frequency of false positives may result from the gain of additional copies of chromosomes, a phenomenon known to occur at high frequency in the F45 background and which often results in loss of colony morphology (Tan *et al.* 2013). Alternatively, other mechanisms such as prion-switching (Holmes *et al.* 2013) or high-frequency mutations (Halme *et al.* 2004) may be responsible for loss of colony morphology in the false positives.

**Figure 1:**
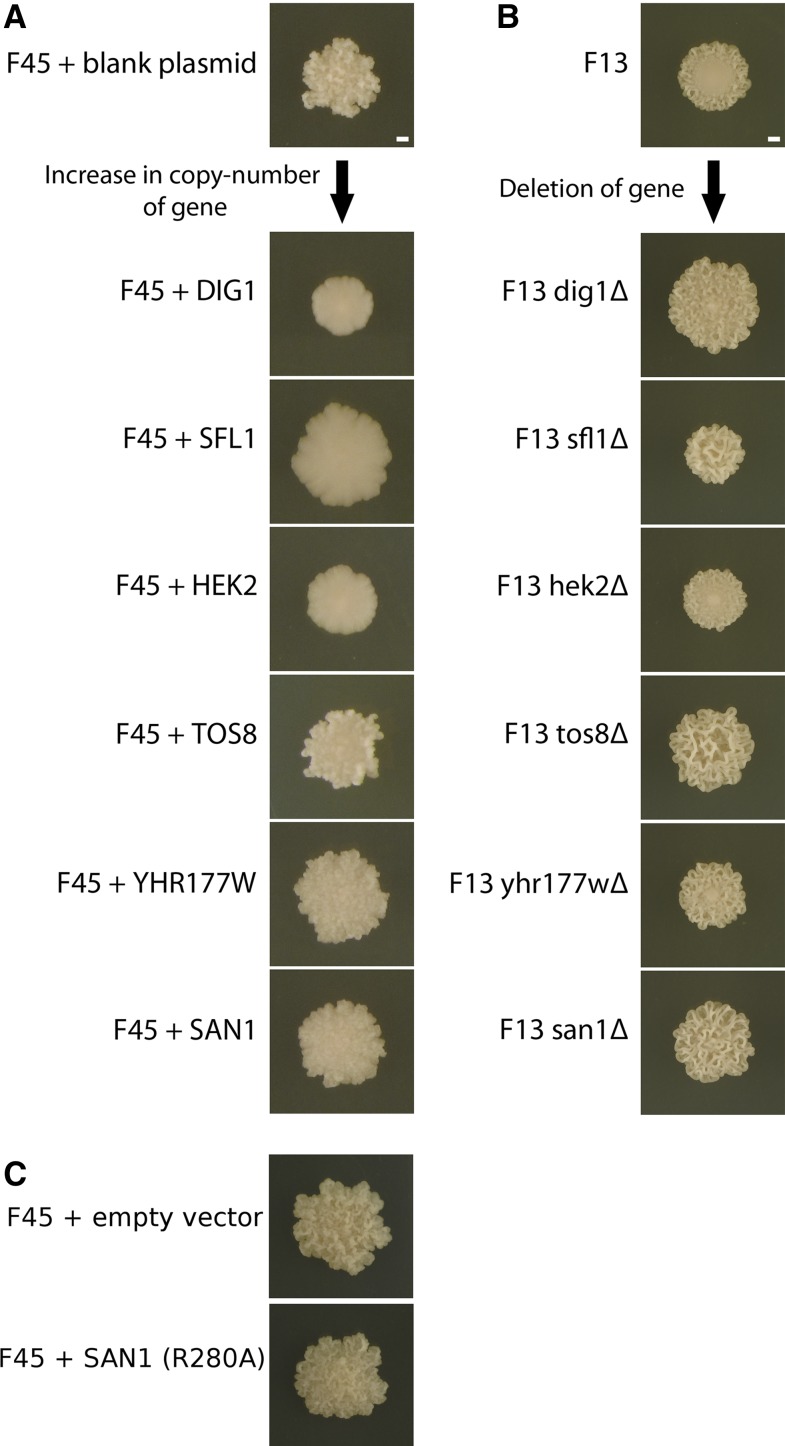
Effects of overexpressing and deleting genes identified in screen for modulators of the fluffy phenotype. (A) Increase in copy number of *DIG1*, *SFL1*, *HEK2*, *ROF1/YHR177W*, *SAN1*, and *TOS8* leads to a reduction in fluffy morphology in strain F45, grown on YPD with 2% glucose. (B) Deletion of *DIG1*, *SFL1*, *HEK2*, *ROF1/YHR177W*, *SAN1*, and *TOS8* leads to an increase in fluffy morphology in strain F13, grown on YPD with 1% glucose. (C) When the catalytically dead *san1-R280A* allele is overexpressed in F45, colonies remain fluffy.

After individual retransformation into F45 and assays of colony morphology, five genes were verified: *SAN1*, *TOS8*, *YHR177W*, *HEK2*, and *SFL1*, which we compared to *DIG1*, identified in our previous screen (Tan *et al.* 2013). The strength of the reduction in colony structure varied from very strong (*DIG1*, *SFL1*, *HEK2*), through intermediate (*SAN1*, *YHR177W*), to weak (*TOS8*). These results (Figure 1A) suggested that modest increases in the copy number of these genes, is sufficient to reduce complex colony morphology to varying degrees (*Materials and Methods*). Because of the weak phenotypes associated with overexpressing some of these genes, we further tested their role in colony morphology by deleting them in a different strain background, F13 (Figure 1B). F13 is a strain from the same cross that produced F45 (Tan *et al.* 2013) and forms colonies that are smooth in the center and structured on the periphery. Our results from F45 suggested that the genes we identified in the overexpression screen(s) suppress colony structure, and consistent with this, deletion of these genes in F13 increased the degree of colony structure in that genetic background (Figure 1B).

Three of the five genes identified in our screen have known links to complex colony phenotypes. *SFL1* encodes a transcriptional repressor of the cell-surface flocculation genes, which are important for complex colony formation (Lo and Dranginis 1998; Guo *et al.* 2000; Conlan and Tzamarias 2001; Halme *et al.* 2004; Granek and Magwene 2010; Voordeckers *et al.* 2012). *YHR177W* is a paralog of *MIT1*, which encodes a transcriptional regulator of pseudohyphal growth (Cain *et al.* 2012), and is an ortholog of *WOR1*, a master regulator of the white-opaque phenotypic switch in *Candida albicans* (Zordan *et al.* 2006). Interestingly, while *YHR177W* has been shown to encode a protein having DNA-binding properties (Cain *et al.* 2012), its effect on colony morphology in the Σ1278b strain background was unclear, with deletion reducing complex colony morphology in one study (Furukawa *et al.* 2011) but having little effect in another (Cain *et al.* 2012). Owing to its identification in our screen, we have given *YHR177W* the name *ROF1* (Regulator Of Fluffy1). *HEK2* encodes an RNA-binding protein which regulates the flocculin *FLO11*, first via regulation of the *ASH1* mRNA transcript, which specifies differential gene expression in mother *vs.* daughter cells, and also through a second, posttranscriptional mechanism (Irie *et al.* 2002; Wolf *et al.* 2010).

To the best of our knowledge, the remaining two genes from our screen have not been previously shown to influence colony morphology. *SAN1* encodes a ubiquitin ligase involved in targeting aberrant nuclear proteins to the proteasome for degradation (Dasgupta *et al.* 2004; Gardner *et al.* 2005). *TOS8* encodes a homeodomain-containing transcription factor whose targets include a statistically significant enrichment for genes involved in bud growth (Horak *et al.* 2002).

For one of these genes, *SAN1*, reagents were available to test whether the catalytic activity of the encoded protein played a role in its effect on colony morphology. To test whether the phenotypic effect of *SAN1* overexpression was dependent on the function of San1 as a ubiquitin ligase, we overexpressed an allele (*san1-R280A*, kind gift of Dr. Richard Gardner) with a single amino acid change in the RING domain that inactivates the protein’s ubiquitin ligase activity without altering protein structure (Fredrickson *et al.* 2013). In contrast to overexpression of fully functional *SAN1*, overexpression of the catalytically dead *san1-R280A* allele had no phenotypic effect, with F45 colonies remaining as fluffy as the vector-alone control (Figure 1C). Therefore, the effect of *SAN1* copy number appears to be mediated through its ubiquitin ligase activity.

### A common transcriptional profile characterizes most of the overexpression strains

Because four of the six genes (including *DIG1*) identified in our overexpression screen are known or putative transcription factors, differences in RNA expression levels could help explain their influence on colony morphology. To investigate this, we began by comparing the mRNA expression patterns of each of the smooth overexpression strains to the fluffy progenitor (F45). Briefly, we isolated total RNA from single colonies growing on solid medium and prepared libraries to measure stranded mRNA using the Illumina TruSeq method (*Materials and Methods*). Each smooth overexpression strain showed a substantial number of genes that were significantly differentially expressed relative to the fluffy F45 vector-alone control (multiple hypothesis corrected *P* < 0.01: *DIG1* = 1976; *HEK2* = 2668; *SAN1* = 1630; *SFL1* = 2385; *TOS8* = 722; *ROF1* = 1567) (Supplemental Material, Table S1). Notably, significant differential expression was seen in the *SAN1* and *HEK2* overexpression strains, despite the fact that these genes do not encode transcription factors.

Comparing the differential expression profiles of these strains identified substantial correlations between the *ROF1*, *HEK2*, *SFL1*, *SAN1*, and, to a lesser extent, *DIG1* overexpression patterns, but little correlation between the strain overexpressing *TOS8* and any of the others (Table 2). In particular, the differential expression profiles of the *HEK2* and *SFL1* strains (*R* = 0.86) and the *SAN1* and *ROF1* strains (*R* = 0.84) were very similar, despite the fact that genes in each pair encode one transcription factor and one protein with a different function. One possible explanation for these correlated pairs is that overexpression of the nontranscription factor leads to increased expression of the transcription factor, resulting in the same transcriptional profile as when the transcription factor is directly overexpressed from the plasmid. However, we found that for both pairs of genes with highly similar expression profiles (*HEK2-SFL1* and *SAN1-ROF1*), the expression level of one gene in the pair was essentially unperturbed by the overexpression of the other (Table 3).

**Table 2:** Correlation (R) between differential gene expression profiles (relative to F45) of overexpression strains

	DIG1	SFL1	HEK2	SAN1	ROF1	TOS8
DIG1		0.70	0.65	0.66	0.49	0.42
SFL1	0.70		0.86	0.74	0.67	0.15
HEK2	0.65	0.86		0.79	0.72	0.09
SAN1	0.66	0.74	0.79		0.84	0.24
ROF1	0.49	0.67	0.72	0.84		0.20
TOS8	0.42	0.15	0.09	0.24	0.20	

**Table 3:** Expression change for each of the genes from our screen in each overexpression strain, relative to F45 (log_2_ fold-change)

	Overexpression Strain					
Gene	DIG1	SFL1	HEK2	SAN1	ROF1	TOS8
DIG1	2.11	−0.11	0.16	−0.03	−0.11	0.10
SFL1	−0.14	2.23	0.10	−0.10	−0.09	0.24
HEK2	−0.07	0.06	1.63	−0.07	0.06	0.06
SAN1	−0.17	0.07	0.19	1.32	−0.05	−0.08
ROF1	−0.06	0.23	0.21	0.05	1.58	0.14
TOS8	−0.04	0.54	0.15	−0.14	−0.02	2.31

To further explore the observed correlations between the RNA expression profiles of many of our overexpression strains, we carried out common factor analysis (*Materials and Methods*). Factor analysis seeks to explain correlations between observed variables (*e.g.*, the individual global expression patterns of our overexpression strains) in terms of a potentially smaller number of unobserved variables called factors or latent variables. Each observed variable is explained as a linear combination of these latent factors plus residual effects unique to the observed variable (including noise).

**Figure 2:**
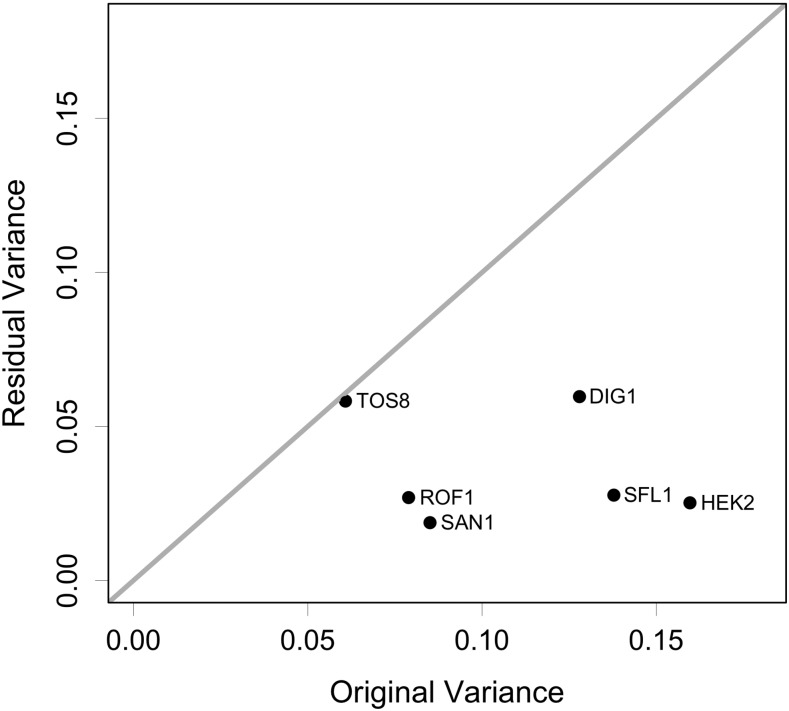
Initial variance in each overexpression strain (variance of log_2_ fold-change relative to F45) and residual variance unexplained by the common factor (using the communality values from factor analysis).

**Figure 3:**
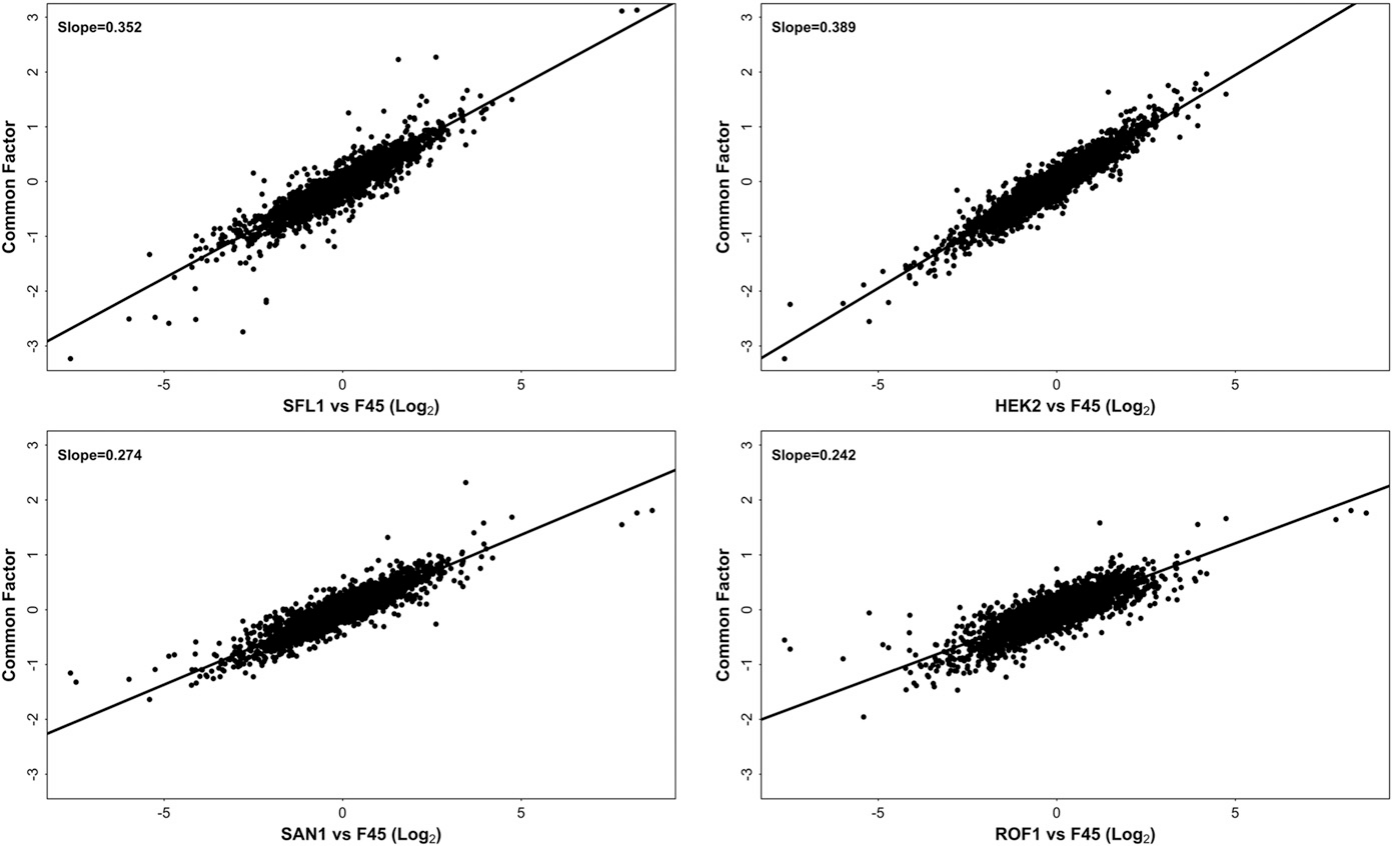
*SFL1*, *HEK2*, *SAN1*, and *ROF1* overexpression profiles (relative to F45) *vs.* the common factor, showing linear regression line and slope.

**Figure 4:**
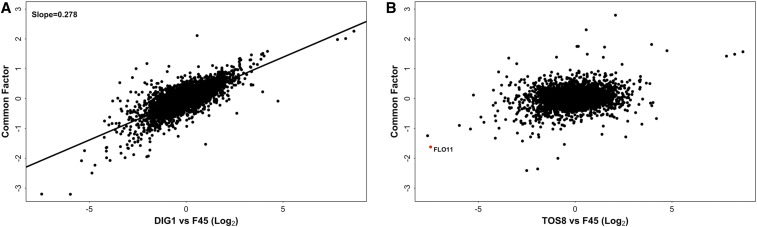
(A) *DIG1* overexpression profile (relative to F45) *vs.* the common factor, with linear regression line and slope. (B) *TOS8* overexpression profile (relative to F45) *vs.* the common factor.

Applying common factor analysis to our transcriptional profiles identified a single strongly significant common factor in the data (Figure S1). This factor explained most of the variance in the *ROF1*, *HEK2*, *SFL1*, and *SAN1* strains and left a similar level of residual variance in each strain, suggesting this residual variance might largely reflect a common level of noise rather than substantial strain-specific effects on expression (Figure 2). The common factor also explained approximately half of the variance in the *DIG1* strain (variance explained = 53%), leaving a substantially higher degree of residual variance for *DIG1* than for the *ROF1*, *HEK2*, *SFL1*, and *SAN1* overexpression strains (Figure 2). As expected from the lack of significant correlation between the *TOS8* and other profiles, the latent factor explained almost none of the *TOS8* differential expression pattern (variance explained = 4%). Taken together, these data suggest that overexpression of *ROF1*, *HEK2*, *SFL1*, and *SAN1* induces a single expression profile (the common factor), although to differing extents, as the slopes of linear regressions between the common factor and the transcriptional profiles of these strains varied from 0.24 for *ROF1* to 0.39 for *HEK2* (Figure 3). In contrast, the effect of *DIG1* overexpression appears to reflect a combination of the common factor and substantial differential expression specific to the *DIG1* strain (Figure 4A), while overexpression of *TOS8* induces a completely distinct profile (Figure 4B). Thus, it appears that a very limited number of transcriptional states are observed in the six overexpression strains: a profile unique to the *TOS8* strain, a single transcriptional state induced to varying extents by the *ROF1*, *HEK2*, *SFL1*, and *SAN1* strains, and a profile unique to the *DIG1* strain, which appears to reflect the common factor along with additional *DIG1*-specific transcriptional effects.

### Potential role of smooth *vs.* fluffy colony environment on gene expression

Because overexpression of all of the genes identified in our genetic screen elicited a common phenotypic response, colonies that are less fluffy than their F45 progenitor, one possible explanation for their similar expression profiles, could be a common response to the smooth *vs.* fluffy colony state or “environment.” For example, complex colonies have a larger surface area to volume ratio than smooth colonies and contain internal cell-free spaces (Sťovíček *et al.* 2012), differences that could alter nutrient and oxygen availability for cells in the two colony types. To test this possibility, we compared the RNA expression profiles of the overexpression strains to our previously published gene expression data for F45 colonies with deletions of two genes, *CIS3* and *FLO11*, which cause F45 colonies to become smooth (although maintaining irregular edges) (Cromie *et al.* 2017). Our previous results suggested that the *CIS3* deletion (*cis3*Δ) provided the cleanest readout of the smooth colony environment, and that this environment does not substantially alter gene expression. Consistent with this result, correlations between the *cis3*Δ and our overexpression profiles were very poor (Table 4), suggesting that the smooth colony environment does not appear to drive the differential gene expression patterns seen in our overexpression strains. We did observe some modest correlations between *flo11*Δ and our overexpression profiles (Table 4). However, *flo11*Δ appears to have effects on the expression of genes involved in colony morphology signaling pathways, rather than just reflecting gene expression associated with the smooth colony state (Cromie *et al.* 2017). These effects are likely to explain the modest correlations between *flo11*Δ and our overexpression profiles.

**Table 4:** Correlation (R) between differential gene expression profiles (relative to F45) of overexpression strains and those of *CIS3* and *FLO11* deletions

	FLO11	CIS3
DIG1	0.36	−0.08
SFL1	0.42	−0.07
HEK2	0.44	−0.05
SAN1	0.29	−0.04
ROF1	0.20	−0.01
TOS8	0.01	0.08

### Shared features of the common factor and TOS8 transcription profiles

Analysis of our overexpression strains identified three independent transcriptional profiles: the common factor, the *TOS8* profile, and the *DIG1* residual profile. Both the common factor and the *TOS8* overexpression profile are associated with a reduction in structured colony morphology. *DIG1* overexpression is also associated with this morphological change but, because the *DIG1* overexpression profile consists of the common factor combined with a *DIG1*-specific residual expression profile, the relationship between the *DIG1* residual profile and colony morphology was not as clear. Therefore, to identify transcriptional changes commonly associated with the fluffy-to-smooth transition, we compared the *TOS8* overexpression profile and the common factor.

First, we functionally characterized the two expression profiles, individually, by looking for gene ontology (GO) term, transcription factor target, and metabolic/regulatory pathway enrichment among the sets of genes significantly (*P* < 0.01, after multiple hypothesis correction) and strongly (>1.5-fold) induced and repressed in each profile (Table S2 and Table S3). This analysis identified several overlapping features of GO term and pathway enrichment. In both profiles, cell periphery genes were overrepresented among induced genes, and genes encoding anchored components of the membrane and reproductive genes were overrepresented among repressed genes. In both the *TOS8* and common factor profiles, MAPK signaling genes were significantly overrepresented among genes repressed in the smooth colonies, as were genes involved in mating. One of the pathways known to induce complex colony morphology is the filamentation MAPK cascade, and the components of this pathway overlap to a large extent with the mating/pheromone-response pathway (Roberts and Fink 1994; Chou *et al.* 2006; Cullen and Sprague 2012).

We hypothesized that the genes consistently upregulated or consistently downregulated in both the common factor and the *TOS8* overexpression profile might include genes involved in the common change in colony morphology associated with both profiles. Consistent with this hypothesis, *FLO11* expression is repressed both in the common factor and in the *TOS8* profile (Figure 4B). Similarly, although there was a very poor correlation between the common factor and the *TOS8* profile across all genes (*R*^2^ = 0.04) (Figure 4B), genes that were significantly (*P* < 0.01, after multiple hypothesis correction) differentially expressed in both profiles tended to show a consistent direction of effect (Fisher’s exact test, 2-tailed: *P* < 2.2e−16). Among 400 such genes, 153 were induced and 173 were repressed in both profiles. In contrast, only 36 genes were induced in the common factor and repressed by *TOS8* overexpression, and only 38 were repressed in the common factor and induced by *TOS8* overexpression.

The genes commonly induced in both the *TOS8* overexpression and common factor profiles were enriched for several GO terms including “transmembrane transport” (GO:0055085; *P* = 8.48e−4), “cell periphery” (GO:0071944; *P* = 1.06e-3), and “extracellular region” (GO:0005576; *P* = 1.26e−3) (Table S4). Similarly, enrichment for several GO terms was seen in the set of genes commonly repressed in both the *TOS8* overexpression and common factor profiles, including “conjugation with cellular fusion” (GO:0000747; *P* = 2.10e−6), “cell periphery” (GO:0071944; *P* = 1.25e−7), and “anchored component of membrane” (GO:0031225; *P* = 4.87e−4) (Table S4). Repressed genes were also enriched for targets of Tec1 (*P* = 3.35e−6), a transcriptional inducer of complex colony morphology that acts in the filamentation MAPK pathway (Chou *et al.* 2006) (Table S4). *TEC1* itself was also one of the genes commonly repressed, as were the flocculin genes *FLO10* and *FLO11* that are known to be effectors of complex colony phenotypes (Lo and Dranginis 1998; Guo *et al.* 2000; Halme *et al.* 2004; Granek and Magwene 2010; Voordeckers *et al.* 2012). These results support the hypothesis that a set of genes important for the fluffy–smooth morphological switch is consistently repressed or induced in both the common factor and *TOS8* overexpression profiles.

### Central role of the filamentation MAPK pathway in the change in colony morphology

The *DIG1* strain has the strongest smooth colony phenotype of any of our overexpression strains (Figure 1A). The transcriptional profile of this strain consists of the common factor plus a residual *DIG1*-specific expression pattern suggesting that the residual profile also contributes to the smooth phenotype. Dig1 is a repressor of the Ste12-Tec1 transcriptional complex (Cook *et al.* 1996; Chou *et al.* 2006), and genes in “MAPK signaling pathway – yeast” (KEGG:04011; *P* = 3.81e−3) and targets of Tec1 (*P* = 1.85e−5) were overrepresented among the genes repressed in the *DIG1*-residual profile (Table S5). Therefore, the residual *DIG1* profile appears to reflect repression of the filamentation MAPK pathway.

As discussed above, many of the genes repressed in both the common factor and the *TOS8* profile are genes in the filamentation MAPK pathway. Consistent with this, many of these genes (80/173) were also downregulated in the *DIG1* residual profile, a highly significant enrichment (Fisher’s exact test, 1-tailed: *P* < 2.2e−16). This further supports the hypothesis that a substantial component of the overlap between the *TOS8* and common factor profiles reflects repression of the filamentation MAPK pathway. In the *DIG1* overexpression strain these genes are repressed as part of the common factor and then further repressed by specific effects of *DIG1* overexpression (*i.e.*, the *DIG1* residual profile). This double signaling through the filamentation MAPK pathway may explain why the *DIG1* overexpression strain has such a strong smooth phenotype (Figure 1A).

### Effects of overexpression strains on noncoding RNAs and nonreference gene transcripts

Finally, we extended our analysis to the expression levels of a large number of noncoding RNAs (ncRNAs) and a small number of genes present in strain F45, but absent from the S288c reference genome (Cromie *et al.* 2017) (*Materials and Methods*). Similar to reference mRNAs, substantial differential expression was observed among these transcripts in all overexpression strains (Table S6). The same pattern of correlations between overexpression strains that was seen with reference genes (Table 2) was also observed with ncRNAs and nonreference mRNAs (Table 5) (Figure S2). This implies that the three underlying transcriptional profiles that we identified from analysis of reference genes extend to include effects on the expression of substantial numbers of nonreference genes and noncoding transcripts.

**Table 5:** Correlation (R) between differential expression profiles (relative to F45) of overexpression strains for ncRNAs and nonreference mRNAs

	DIG1	SFL1	HEK2	SAN1	ROF1	TOS8
DIG1		0.72	0.75	0.62	0.49	0.48
SFL1	0.72		0.87	0.65	0.59	0.33
HEK2	0.75	0.87		0.71	0.62	0.29
SAN1	0.62	0.65	0.71		0.70	0.38
ROF1	0.49	0.60	0.62	0.70		0.37
TOS8	0.48	0.33	0.29	0.38	0.37	

## Discussion

### Morphology change as a regulated switch between transcriptional states

Several mechanisms have been shown to govern the stable switching between structured/biofilm and nonstructured colony morphologies in *S. cerevisiae*. These include high-frequency mutations (Halme *et al.* 2004) and prion-based mechanisms (Holmes *et al.* 2013) as well as aneuploidy, specifically the gain and loss of additional copies of whole chromosomes (Tan *et al.* 2013). Notably, several of these mechanisms are transcriptional in nature. In the F45 strain used in this study, overexpression of the Dig1 transcriptional repressor contributes to the fluffy-smooth switch induced by a chromosome XVI disome (Tan *et al.* 2013). In another case, high-frequency mutations that inactivate the Ira1 and Ira2 Ras-activating proteins (Halme *et al.* 2004) cause changes in colony morphology through the Ras-cAMP-PKA pathway which regulates the Sfl1 and Flo8 transcription factors (Rupp *et al.* 1999). Similarly, switching between the prion and nonprion forms of the Mot3 transcription factor produces a colony morphology switch (Holmes *et al.* 2013).

One of the best-characterized phenotypic switches regulated by a transcriptional circuit in fungi is the white to opaque switch in *C. albicans* (reviewed in Huang 2012; Soll 2014). The opaque state is necessary for mating, while white cells can form structured biofilms that are highly impermeable to drugs and components of the immune system. Each of the two states appears to be adaptive for survival in different host niches, with white cells being favored in systemic infection models and opaque cells showing improved ability to colonize the skin. A transcriptional circuit with two stable states underlies the white and opaque phenotypes, with white being the default state. Because switching between states leads to the differential expression of a large number of genes, each state is associated with a specific expression profile. Interestingly, *WOR1* (a homolog of *S. cerevisiae ROF1*) is the master regulator of the white-opaque switch and ectopic expression of *WOR1* can drive an entire population of white cells to become opaque (Huang *et al.* 2006).

Similar to the effect of overexpressing *WOR1* in *C. albicans* white-opaque switching, we propose that overexpression of most of the genes identified in our study produces a common change in phenotype and gene expression that replicates an adaptive, regulated switching mechanism. That is, some aspect(s) of the regulated switching between the fluffy and smooth transcriptional states produces an adaptive change in colony morphology, and the expression profiles that we observe reflect transcriptional states that *S. cerevisiae* may access in response to environmental conditions favoring the biofilm or nonbiofilm states of the microbial community. We previously demonstrated that the smooth and fluffy (biofilm) states of the F45 background used in this study are each adaptive in different environmental conditions (Tan *et al.* 2013).

The regulated transcriptional switching model makes several key predictions. First, regulatory genes should exist that control the process and perturbation of these genes could trigger the phenotypic switch between the fluffy and smooth colony morphologies, mimicking signaling through those genes. Second, perturbation of regulatory genes acting in the same pathway should produce the same transcriptional state. Third, the transcriptional response should alter the activity of downstream pathways that effect the morphological change. Our data are consistent with each of these expectations.

### Regulatory repressors of biofilm formation

The six genes that caused fluffy-to-smooth transitions in our overexpression screens all encode known or predicted regulatory proteins, with four being transcription factors. In contrast, we did not identify any genes that appear to have mechanistic roles in colony morphology, such as cell-surface proteins, despite the fact that deleting such genes individually (*e.g.*, *cis3*Δ and *flo11*Δ) is sufficient to cause a fluffy-to-smooth transition in the same genetic background (Cromie *et al.* 2017). Furthermore, the genes that we identified appear to be components of (or to interact with) regulatory pathways that repress the complex colony phenotype. In fact, *DIG1* and *SFL1* encode known transcriptional repressors of genes needed for complex colony formation, and act in the filamentation MAPK and Ras-cAMP-PKA signaling pathways, respectively (Conlan and Tzamarias 2001; Chou *et al.* 2006; Granek and Magwene 2010).

Our study also identified a phenotype for genes that were previously characterized only at the level of biochemical activity (known or predicted). This was true for the putative transcription factor Rof1, which we named based on its phenotype in this study, and the ubiquitin ligase San1, neither of which had been linked to colony morphology prior to our study. Previous characterization of San1 had identified a number of target proteins (Gardner *et al.* 2005; Rosenbaum *et al.* 2011), and the phenotype recognized here may help identify specific pathways that are regulated by this activity. This may also be true for *HEK2*, which encodes a protein that has been shown to bind to a large number of mRNAs (Hogan *et al.* 2008).

### Convergence on a common transcriptional state

We expected that perturbation of genes operating in the same regulatory pathway would produce the same transcriptional response. Overexpression of four of the six genes that we identified (*SFL1*, *SAN1*, *ROF1*, and *HEK2*) produced a single transcriptional state, the common factor, consistent with them operating in a single transcriptional regulatory pathway. This common factor is also a large component of the transcriptional profile observed when a fifth gene, *DIG1*, is overexpressed.

The “single regulatory pathway” identified by these genes is probably better understood as a signaling network, as it encompasses both the cAMP-PKA pathway (including *SFL1*) and the filamentation MAPK pathway (including *DIG1*) (Vinod *et al.* 2008). Cross-talk between these pathways is known to exist (Mosch *et al.* 1999; Borneman *et al.* 2006) allowing signal integration to occur (Cullen and Sprague 2012). Such a signaling network could integrate a range of environmental signals to produce a transcriptional response, and ultimately a phenotypic change. This model can explain how increasing the expression of genes that do not directly regulate gene expression, such as *HEK2* and *SAN1*, can produce a strong transcriptional response, and one shared with transcription factors such as *SFL1* and *ROF1*. In this case, *SFL1* and *ROF1* represent (complex morphology repressing) transcriptional nodes in the signaling network, while *HEK2* and *SAN1* might regulate elements of the signaling pathways at the RNA- and protein-abundance levels. For these four genes, the signal produced by overexpression would, after signal integration across the network, have the same final effect in terms of which transcriptional state the cell/colony adopts. That is, the transcriptional state corresponding to the common factor may be one that emerges from any one of a range of perturbations.

In contrast to the other genes in our study, overexpression of *TOS8* did not induce the common factor transcriptional state. This result suggests that Tos8 operates in a different regulatory pathway than the products of the other genes. This, in turn, suggests that the *TOS8* overexpression profile may represent a distinct transcriptional state that cells can adopt under environmental conditions that differ from those producing the common profile.

### Genes important for the smooth–fluffy morphological switch

The common factor and *TOS8* transcriptional profiles are essentially uncorrelated and only a small proportion of genes are significantly differentially expressed in both. However, among the overlap between these two profiles, there is a statistically significant overrepresentation of genes showing differential expression with the same direction of effect, *i.e.*, induced or repressed in both profiles, compared to genes that have opposite directions of effect. This result suggests the existence of a differentially expressed subset of genes that are involved in the common fluffy–smooth morphological change associated with both profiles. Because the overexpression strains appear to be operating through gene expression regulatory mechanisms, the commonly differentially expressed genes may represent modules of coregulated genes that respond as a group to environmental signals.

A high proportion of the genes commonly repressed in both the *TOS8* and common factor profiles were also significantly repressed in the residual *DIG1*-expression profile, *i.e.*, the gene expression changes seen in the *DIG1* overexpression strain, after accounting for the effect of the common factor. It therefore appears that *DIG1* and the filamentation MAPK pathway are particularly important in control of the critical gene module(s), repressing genes whose expression is required for fluffy colony formation.

## Supplementary Material

Supplemental material is available online at www.g3journal.org/lookup/suppl/doi:10.1534/g3.117.042440/-/DC1.
