# Supplementary figures and images for: Transcriptional Profiling of Biofilm Regulators Identified by an Overexpression Screen in Saccharomyces cerevisiae
Source: G3 (Bethesda). 2017 Jul 3;7(8):2845–54. doi: 10.1534/g3.117.042440 (PMC5555487; doi:10.1534/g3.117.042440)

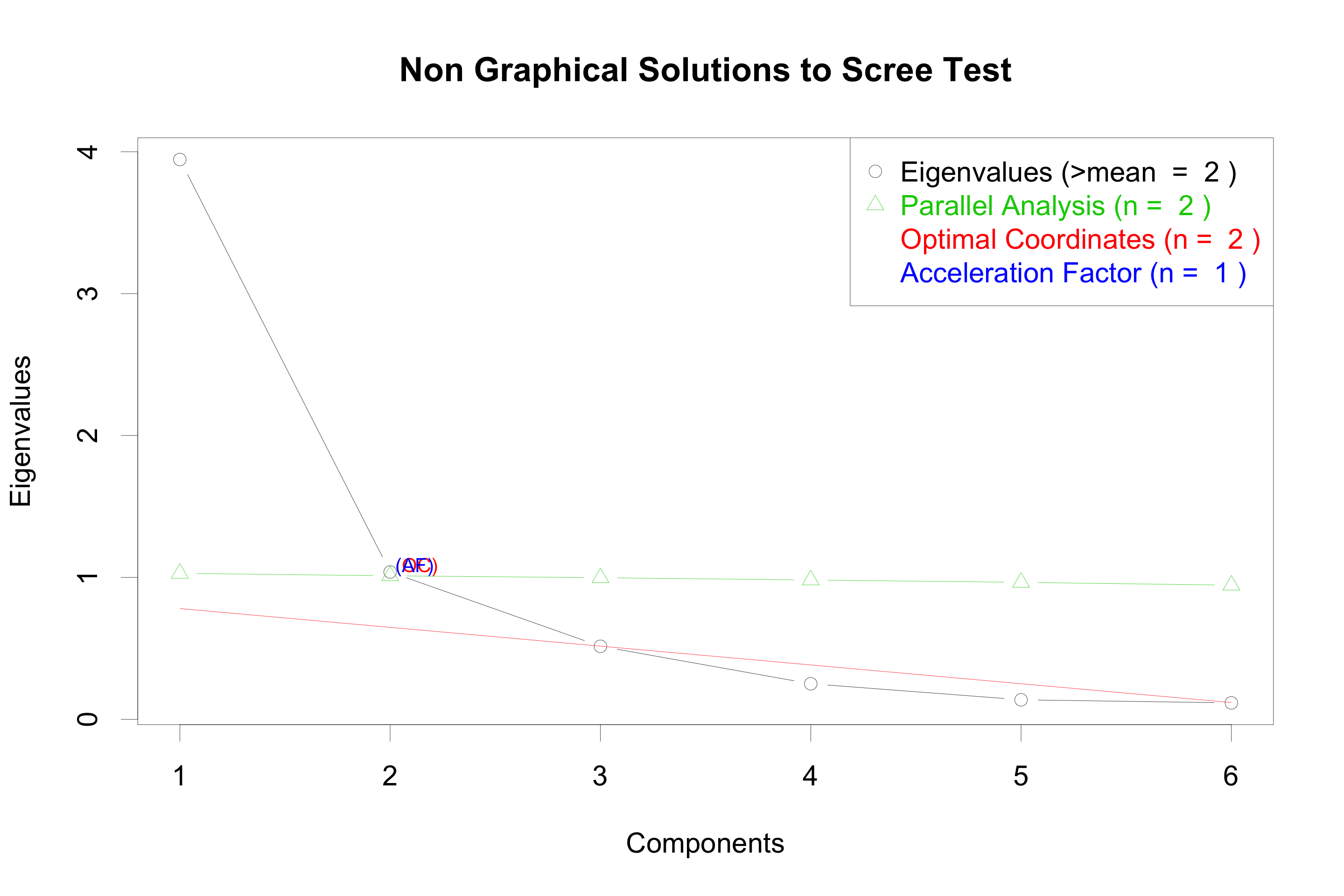

Supplement: Supplementary file 1 [file 2845FigureS1.tiff]

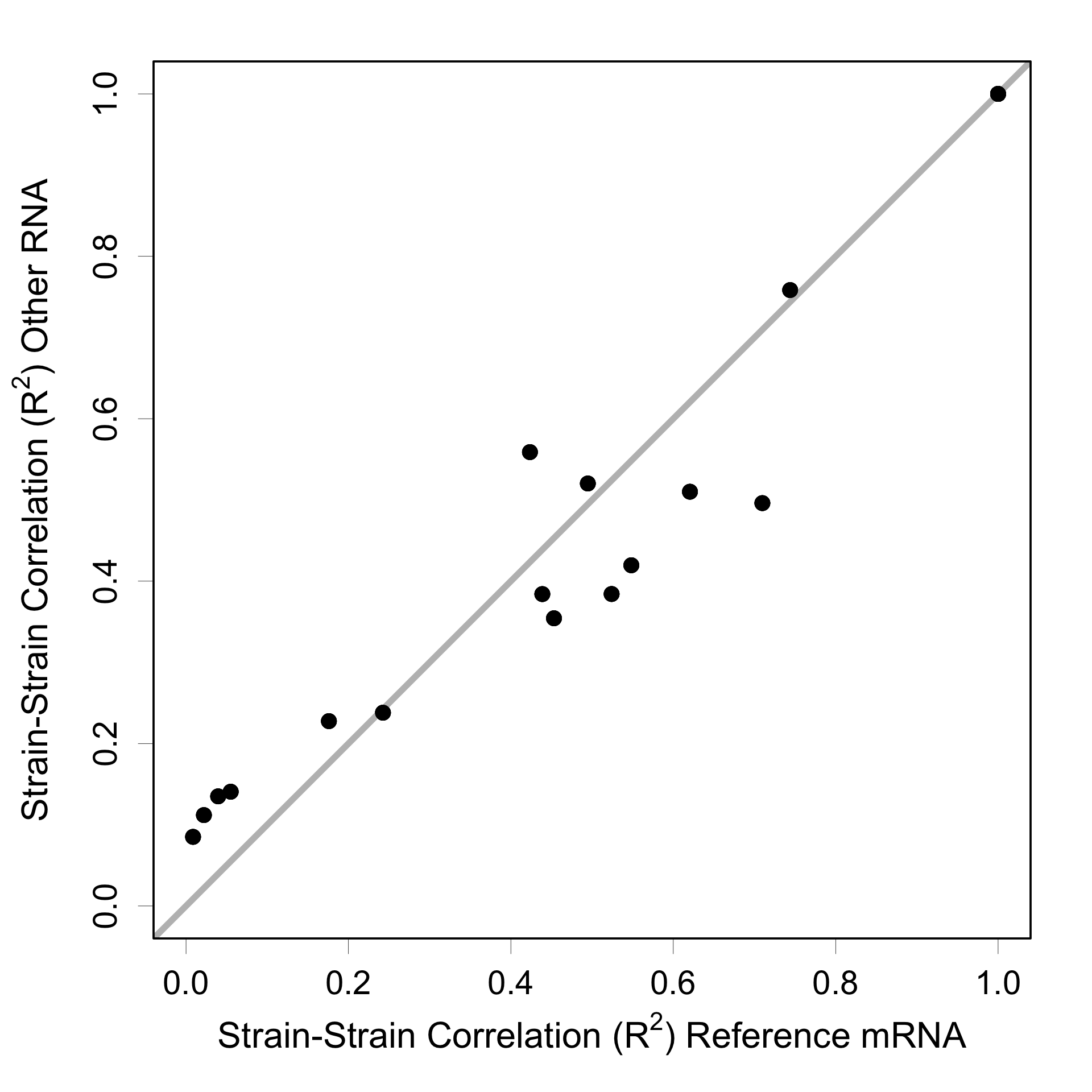

Supplement: Supplementary file 2 [file 2845FigureS2.tiff]
